# *Anagnorisma chamrani* sp. n. (Lepidoptera, Noctuidae) from Iran

**DOI:** 10.3897/zookeys.317.5515

**Published:** 2013-07-15

**Authors:** Peter Gyulai, Mohammad Mahdi Rabieh, Ali Asghar Seraj, Laslo Ronkay, Mehdi Esfandiari

**Affiliations:** 1 Mélyvölgy u. 13/A, H-3530 Miskolc, Hungary; 2 Department of Plant Protection, College of Agriculture, Shahid Chamran University of Ahvaz, Ahvaz, Iran; 3 Department of Zoology, Hungarian Natural History Museum, H-1088 Budapest, Baross u. 13

**Keywords:** *Anagnorisma*, Noctuidae, new species, Iran

## Abstract

A new *Anagnorisma* species, *Anagnorisma chamrani* **sp. n.**, is described from Binaloud Mountains of Khorasan-e-Razavi province in north-eastern Iran, and compared with its sister species, *Anagnorisma eucratides* (Boursin, 1960). The adults, and male and female genitalia of both species are illustrated in 11 figures. The genus *Anagnorisma* is recorded for the first time for the fauna of Iran.

## Introduction

The genus *Anagnorisma* was erected by Ronkay and Varga (1999), together with two further genera of the *Eugnorisma* Boursin, 1946 generic complex, *Protognorisma* Ronkay & Varga, 1999 and *Schizognorisma* Ronkay & Varga, 1999. These three genera represent the more ancient lineages of this clade; their species are generally distributed in the wide sense Himalayan region. The detailed comparison of the three genera, and the main differential features between the sister genera *Anagnorisma* and *Schizognorisma*, are given in the original descriptions (Ronkay and Varga op. cit.).

The genus *Anagnorisma* was known to comprise four taxa, *Anagnorisma eucratides* (Boursin, 1957), *Anagnorisma goniophora* (Hacker, Ronkay & Varga, 1990), *Anagnorisma glareomima* (Varga & Ronkay, 1991) and *Anagnorisma zakaria* Ronkay & Varga, 1999. The fifth species, the most westerly distributed member of the genus, *Anagnorisma chamrani* sp. n., is described below.

## Material and methods

The material examined was collected in late summer 2012 in Khorasan-e-Razavi province located in north-eastern Iran, using 8 watt black light UVB tubes. The genitalia of both sexes were dissected, stained, and mounted following the standardised way. The adults were photographed by Gábor Ronkay, the genitalia slides by Zoltán Soltész and Tibor Csővári; the digital images have been adjusted and edited by Gábor Ronkay.

## Systematic Account

### Subfamily Noctuinae Latreille, 1809
Tribe Noctuini Latreille, 1809
Subtribe Noctuina Latreille, 1809

#### Anagnorisma chamrani

Gyulai, Rabieh & Ronkay sp. n.

urn:lsid:zoobank.org:act:B912288F-2152-4743-B131-AAA3C08F7D48

http://species-id.net/wiki/Anagnorisma_chamrani

Figs 3 , 4 , 8 – 10

##### Type material.

**Holotype.** female, Iran, prov. Khorasan-e-Razavi, Binaloud, 2507 m, 36°28'56"N, 59°46'17"E, 10.IX.2012; slide No. 3145 PGY. The holotype is deposited in the collection of P. Gyulai, later to be deposited in the Hungarian Natural History Museum, Budapest, Hungary

##### Paratype.

Male, with the same data as the holotype; slide No. 3144 PGY (coll. P. Gyulai, Miskolc).

##### Diagnosis.

**Figure 1.**
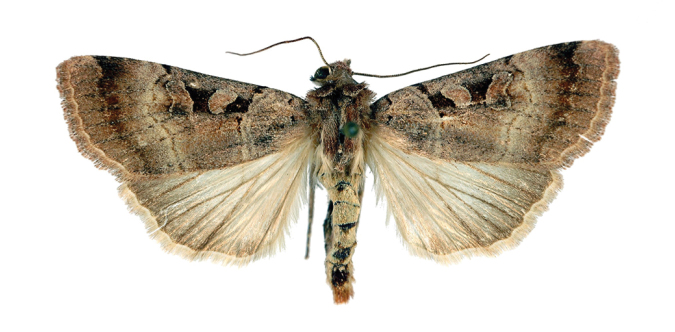
*Anagnorisma eucratides* female, HT.

**Figure 2.**
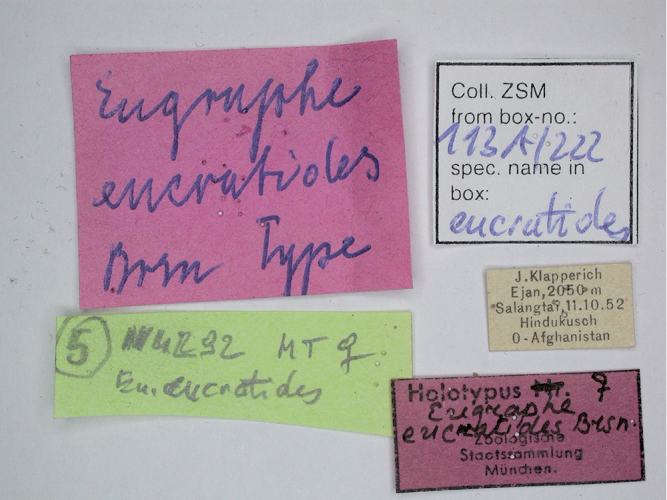
*Anagnorisma eucratides* female, HT labels.

*Anagnorisma chamrani* is the sister species of *Anagnorisma eucratides* (Boursin, 1957), which is only known from eastern Afghanistan at altitudes of 2050 to 2450 m of the Hindu Kush Mountains.The main external differential features, in comparison with *Anagnorisma eucratides* (Figs 1, 2), are the following: thoracic pubescence, ground colour of forewing and filling of stigmata more unicolorous, not brownish red as in *Anagnorisma eucratides*; antemedial line oblique, somewhat zigzagged; postmedial line more crenellate, both of them have a finer black-marked definition; subterminal line less wavy; hindwing darker greyish; cilia pale pinkish. The new species is also similar to the Pakistani *Anagnorisma goniophora*, but the antemedial line is more zigzagged, the postmedial line is less evenly arched, being terminally oblique and not perpendicular to the inner edge of the forewing, the subterminal line is more wavy in the upper half and more conspicuously ochreous. The two other members of *Anagnorisma* are less similar to the new species; *Anagnorisma chamrani* differs from *Anagnorisma glareomima* by its darker and more elongated wings, larger stigmata and the different configuration of the stigmata and the black intermaculation; finally, it cannot be confused with the conpicuously different, orange-brown coloured *Anagnorisma zakaria*. Wingspan 34–35 mm.The configuration of the genitalia of both sexes indicates the close relationship between *Anagnorisma eucratides* and *Anagnorisma chamrani*. In the male genitalia (Figs 6, 7, 9, 10), the dorsal costa of the valva of *Anagnorisma chamrani* is more extended medially, so the costal and dorsal margins are less parallel than in *Anagnorisma eucratides*; the valval apex is evenly rounded, more prominent dorsally than ventrally (it is the opposite in *Anagnorisma eucratides*); the apical lobe and the ventral extension are smaller; the vinculum is somewhat longer; the aedeagus is slightly curved ventrad; the dorsal and ventral carinal plates are narrow, crest-like, long and strongly sclerotised, the ventral plate terminates in an obtuse, small peak (the ventro-lateral carinal plate of *Anagnorisma eucratides* is markedly stronger, bearing a conspicuous, strong thorn).

The male genitalia of *Anagnorisma chamrani* are strikingly dissimilar from those of the externally similar *Anagnorisma goniophora*, especially the broader valvaof *Anagnorisma chamrani*, particularly its distal part and the assemblage of the valval apex; the much longer, arched harpe and the longer, crest-like carinal plates. The diagnostic features, in comparison with *Anagnorisma zakaria*, are the much longer, arched harpe, the considerably shorter (about half as long) ventral valval extension and the longer and not extended, crest-like ventral carinal plate in *Anagnorisma chamrani*.

In the female genitalia, *Anagnorisma chamrani* (Fig. 8) differs from *Anagnorisma eucratides* by its narrower but higher, more sclerotised, asymmetrically subquadrangular antrum with U-shaped postero-medial incision; longer ductus bursae with less elbow-like lateral projection and the almost twice as long, more prominent, conical appendix bursae. Comparing with *Anagnorisma glareomima* and *Anagnorisma zakaria*, the most conspicuous difference is the shape of ductus bursae: *Anagnorisma chamrani* has an almost evenly-broad ductus bursae with an asymmetrical medial constriction in one side and elbow-like lateral projection on the opposite side, whereas the two other species have a funnel-like ductus bursae.

**Figure 3.**
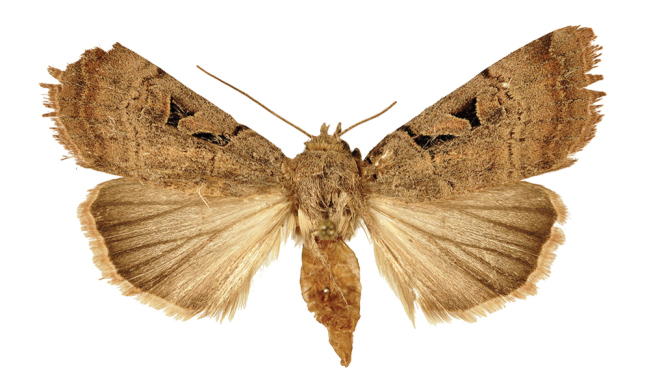
*Anagnorisma chamrani* female, HT.

**Figure 4.**
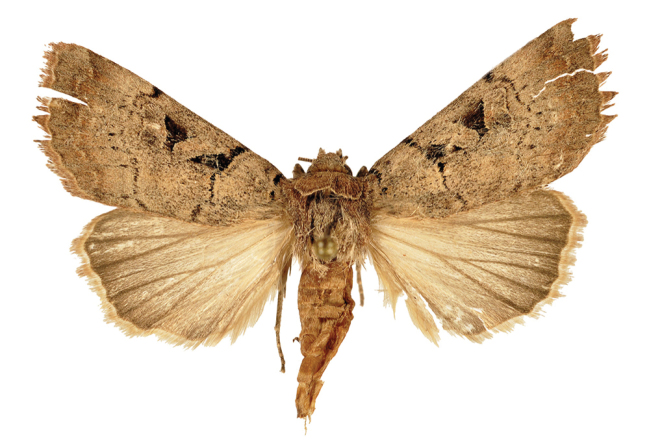
*Anagnorisma chamrani* male, PT.

**Figure 5.**
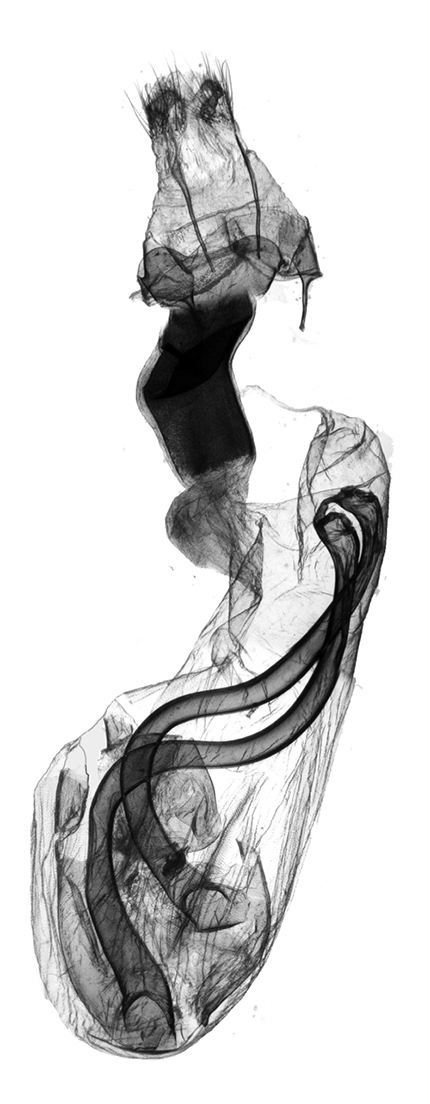
*Anagnorisma eucratides* female genitalia ZSM N4212 HT.

**Figure 6.**
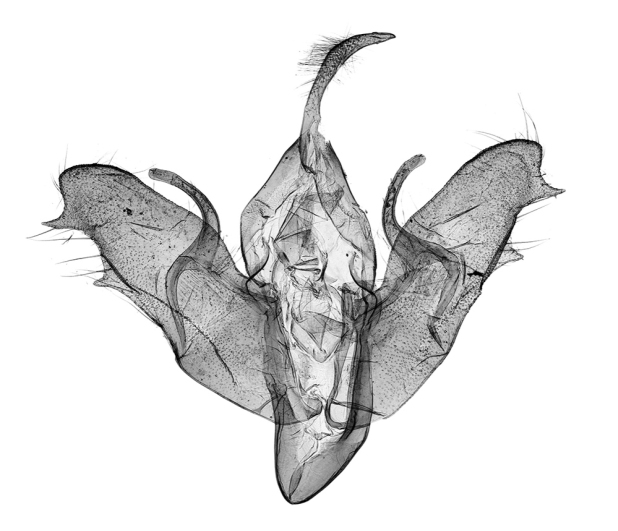
*Anagnorisma eucratides* male genitalia, clasping apparatus PT.

**Figure 7.**
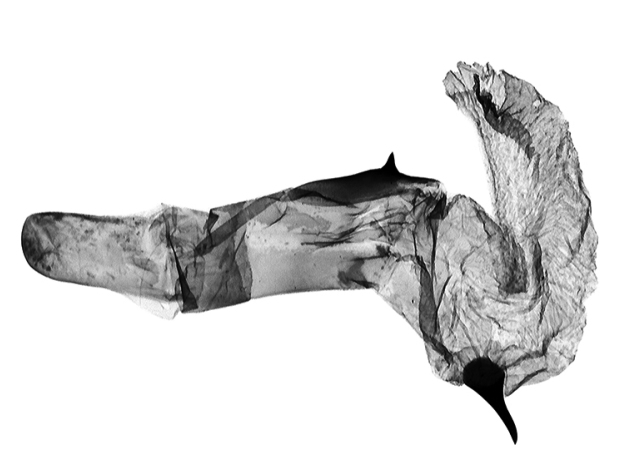
*Anagnorisma eucratides* male genitalia, aedeagus PT.

**Figure 8.**
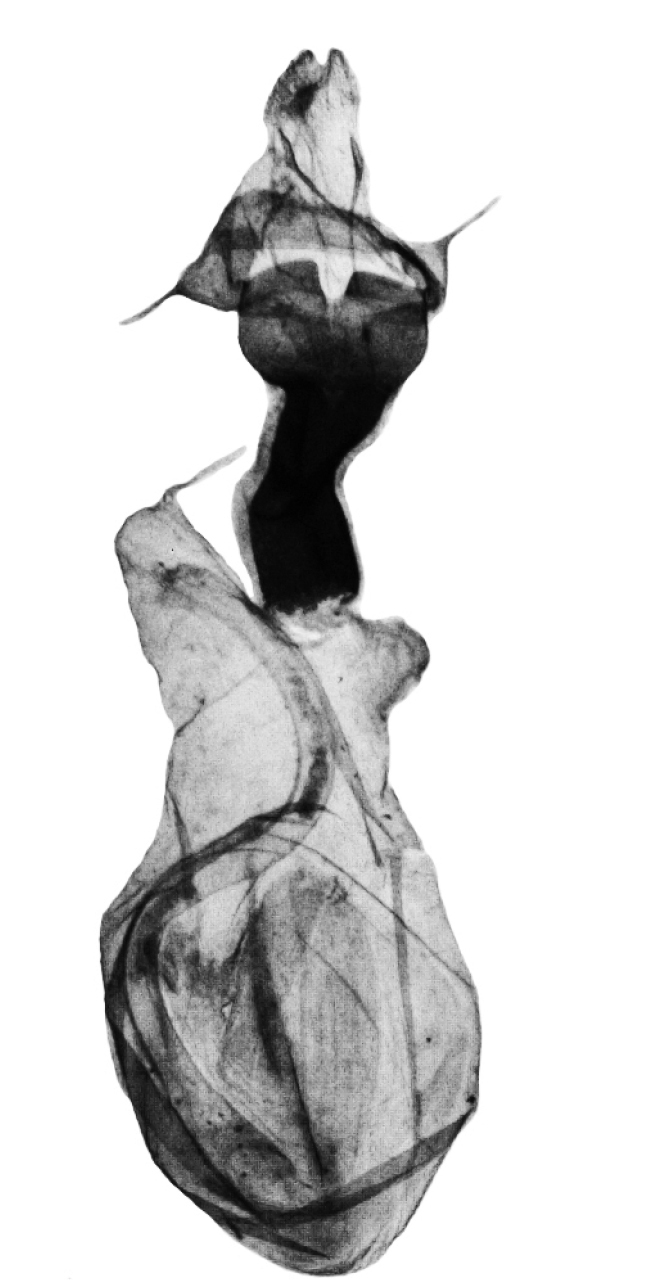
*Anagnorisma chamrani* female genitalia, HT.

**Figure 9.**
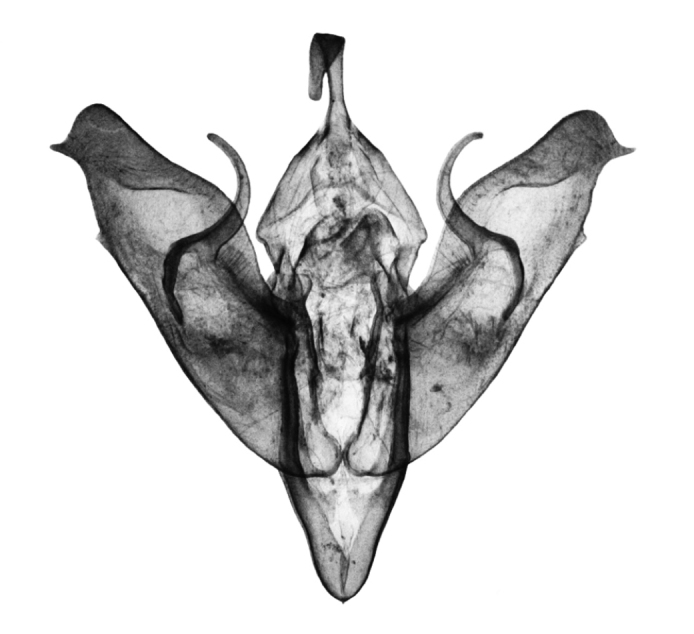
*Anagnorisma chamrani* male genitalia clasping apparatus PT.

**Figure 10.**
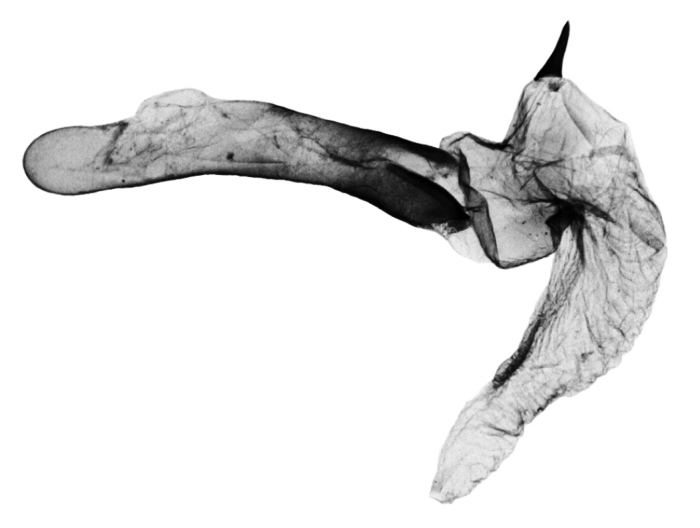
*Anagnorisma chamrani* male genitalia aedeagus PT.

##### Distribution.

**Figure 11.**
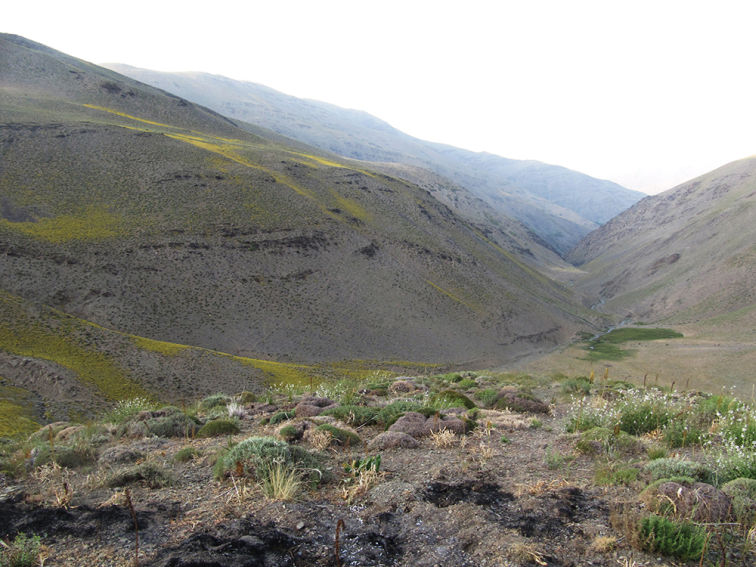
Habitat of the type locality of the new species.

The new species is known only from the type locality, the higher Binaloud Mountains (Fig. 11). The dominant species in the vegetation of the habitat are Mountain Sainfoin, Wild almond (*Amygdalus scoparia*), Downy brome (*Bromus tectorum*); the other mentionable plants are *Bromus*, *Artemisia* and *Astragalus* spp. The adults were attracted to light early on a cold night in September. The early stages are unknown.

##### Etymology.

The new species is named in honour of the martyr Dr. Mostafa Chamran (1932–1981). In 1982 the Jundi Shapur University was renamed to Shahid Chamran University after the martyrdom of Dr. Chamran, an outstanding Iranian warrior in the 8 years Iran–Iraq war. The university was closed in those days due to the war conditions in the area.

## Supplementary Material

XML Treatment for Anagnorisma chamrani
